# Suspected Adverse Drug Reactions in Pediatric Cancer Patients in China: An Analysis of Henan Province Spontaneous Reporting System Database

**DOI:** 10.3389/fonc.2021.807171

**Published:** 2021-12-20

**Authors:** Zhiming Jiao, Zhanchun Feng, Ziqi Yan, Jinwen Zhang, Gang Li, Ganyi Wang, Qianyu Wang, Da Feng

**Affiliations:** ^1^ School of Medicine and Health Management, Tongji Medical College, Huazhong University of Science and Technology, Wuhan, China; ^2^ Department of Pharmacy, Tongji Hospital, Tongji Medical College, Huazhong University of Science and Technology, Wuhan, China; ^3^ Medical Products Administration and Center for Adverse Drug Reaction (ADR) Monitoring of Henan, Zhengzhou, China; ^4^ College of Public Administration, Huazhong University of Science and Technology, Wuhan, China; ^5^ School of Pharmacy, Tongji Medical College, Huazhong University of Science and Technology, Wuhan, China

**Keywords:** pharmacovigilance, drug monitoring, adverse drug reaction reporting systems, pediatric, medical oncology

## Abstract

**Introduction:**

Adverse drug reactions (ADRs) in pediatric cancer patients have not yet received due attention in the world. Antineoplastic drugs are frequently related to ADRs. Few studies focus on the ADR and the intervention measures in pediatric cancer patients.

**Methods:**

ADR reports submitted to Henan Adverse Drug Reaction Monitoring Center from 2016 to 2020 for individuals aged from birth to 17 years (including 17 years) were included. Data were analyzed with respect to gender, age, disease types, past history of ADR, occurrence time of ADR, polypharmacy, route of administration, off-label drug use, name of suspected drugs per ADR report, and severity of ADR reports.

**Results:**

A total of 431 ADR reports related to antineoplastic drugs in pediatric patients were collected, 31.55% were serious ADRs (SADRs). The median age of patients was six years (inter quartile range, IQR: 3-11), the age groups with higher reporting rates were concentrated in 1-3-year-olds (130). Past history of ADR, occurrence time of ADR and polypharmacy were statistically associated with SADR. Myelosuppression was the most frequent ADR (15.55%), cytarabine was the most frequent drug (26.22%). The signal mining method produced 14 signals, three signals were off-label ADRs.

**Conclusions:**

This study described the characteristics of ADRs in pediatric cancer patients. By conducting signal mining method, three off-label ADRs need further study. We should pay more attention to these ADRs and develop relative management strategies. More researches are needed to achieve a better understanding of the characteristics of ADRs in pediatric cancer patients of China.

## Introduction

As a leading cause of death, cancer is a growing public health problem worldwide (1). Approximately 18.1 million new cancer cases and 9.6 million cancer deaths were recorded in 2018 (2). Cancers rarely occur before the age of 20 (3). However, more than 1,000 children are diagnosed with cancer every day globally, and the disease remains the leading cause of death in children and adolescents (3). Approximately 84% of childhood cancers occur in low-income and middle-income countries (4).

As the developing country with the highest population in the world, China has a tough condition of cancer in childhood and adolescents (5). A growing body of literature has investigated childhood and adolescent cancer in China. The first national childhood cancer profile in China was reported in 2015 (6). It provided nationwide incidence, mortality, and temporal trends for childhood cancer from 2003 to 2005. A recent study assessed the childhood cancer incidence patterns from 2000 to 2015 and showed that cancer incidence has increased significantly in children and adolescents in China (5).

Childhood cancer incidence is on the rise worldwide. Pediatric patients have to face problems due to adverse drug reactions (ADR), which are harmful or unpleasant reactions resulting from an intervention related to the use of a medicinal products (7). A meta-analysis of the incidence of ADRs in hospitalized patients showed that the overall incidence of serious ADRs was 6.7%, and fatal ADRs was reported in 0.32% of hospitalized patients (8). A systematic review showed that the overall incidence of ADRs in hospitalized children was 9.53%, and severe reactions accounted for 12.29% of the total. Moreover, 39.3% of the ADRs that caused hospital admissions were fatal reactions (9). A previous research has suggested that some subgroups of children and adolescents are at greater risk of developing ADRs, particularly pediatric cancer patients (10).

However, few studies focused on the ADR in pediatric cancer patients. Mascolo (11) analyzed the safety profiles of antineoplastic drugs in Italy and described the off-label use in pediatric patients. Amaro-Hosey (12) assessed the incidence and characteristics of ADRs in a pediatric oncohematological population in Spain. Research on intervention measures in pediatric cancer patients still has gaps. Studies about ADRs in pediatric cancer patients are limited in China.

This study aimed to analyze serious and normal ADR reports and identify safety signals in children, which improves the safety profile of pediatric cancer patients in clinical practice.

## Methods

### Study Design and Setting

We carried out a cross-sectional study of pediatric cancer patients with suspected ADRs based on the Henan Provincial Adverse Drug Reaction Monitoring Center, China. We designed to analyze different variables in the reports — mainly the difference between serious ADRs and normal ADRs.

### Participants

The following inclusion criteria were used: 1) reported between 2016 and 2020; 2) reports of certain, probable, and possible relationships of drugs; 3) drugs suspectedly associated with ADR was antineoplastic drug; 4) age of 0–17.

The exclusion criteria were as follows: 1) reports before 2016 and after 2020; 2) duplicate records; 3) missing critical information, particularly drug name, and specific records of ADR; 4) unreasonable records, such as records older than 120 years, record that does not match the age, and negative number pertaining to the occurrence time of ADRs.

### Variables

Gender, age, disease types, past history of ADR, occurrence time of ADR, polypharmacy, route of administration, off-label drug use, name of suspected drugs per ADR report, and severity of ADR reports (serious, normal) were collected. The ADRs and clinical manifestations were organized according to the Medical Dictionary for Regulatory Activities (MedDRA) (version 24.0). ADR reports with antineoplastic drug were identified from the 2nd level of the Anatomical Therapeutic Chemical (ATC) Classification System (L01-antineoplastic agents). The generic names of drugs were standardized and coded according to the catalog of generic names for common prescription drugs. The catalog was issued by the Ministry of Health of China in 2007. The most common definition for polypharmacy in children included the use of two or more medications (13). However, the use of multiple therapeutic classes of medications is likely warranted in “complex chronic conditions” such as childhood cancer (14). Thus, our study also adopted the more conservative definition of polypharmacy (five or more medications). Off-label drug use was classified into the following categories: defined as the administration of a prescription drug outside the age range for which the product was licensed; defined as the prescription of a drug for therapeutic indications that were not licensed. The severity of ADR was classified by the reporters and included in the database. Based on the Reporting and Monitoring Administration Measure on ADR issued by the Ministry of health of China (15), the “Serious ADRs” (SADRs) was defined as and the other cases were regarded as “Normal ADRs”: 1. results in death; 2. is life-threatening; 3. carcinogenesis, teratogenesis and congenital disabilities; 4. results in persistent or significant disability/incapacity; 5. require inpatient hospitalization or prolongation of existing hospitalization; 6. leading to other important medical events, such as the situations listed above may occur without treatment.

### Data Sources

We classified and analyzed the Henan Provincial Adverse Drug Reaction Monitoring Center data from 2016 to 2020. The center is subordinate to the National Center for ADR Monitoring, China. These data were reported by Henan medical institutions, enterprises, and the public. Because the data generated from the spontaneous report system (SRS), we cannot get ADR incidence rates as the true extent of drug use was unknown, so all the data in the study were frequency of reports.

### Study Size

A total of 394,037 initial data were obtained. According to the inclusion and exclusion criteria, 431 records were retained. To prevent the repetitive analysis of some reports, we selected one of the main adverse reactions included.

### Statistical Methods

The demographic characteristics, disease types, past history of ADRs, occurrence time of ADRs, polypharmacy, route of administration, and off-label drug use in the report were subjected to descriptive analysis, Fisher exact test and Chi-square test. All data analyses were performed using SPSS 24.0 (IBM Corp. Armonk, NY). A p-value of less than 0.05 was considered statistically significant.

The number of ADRs of each drug was sorted for ADR signal mining, which quantified the qualitative nature of the relationship between drugs and ADRs. In ADR signal mining, the reporting odds ratio (ROR), proportional reporting ratio (PRR), and comprehensive standard method (MHRA) were adopted as measures of disproportionality, which are generally used in detecting the imbalance of target events compared with other events in the database (16, 17). When the target drug event combination (DEC) frequency was significantly higher than the background frequency and reached the threshold, a signal was considered generated. The strength of the association between drugs and ADRs was expressed as the ROR and PRR with 95% confidence intervals (CIs). We listed the equations and criteria for the three algorithms in **Table 1**.

**Table 1:** Formulas and criteria for generating signals of ROR, PRR and MHRA.

Method	Formula	Criteria and threshold
ROR	$\text{ROR}=\frac{\left(\text{a}/\text{c}\right)}{\left(\text{b}/\text{d}\right)}$ $95\%\mathrm{Cl}=e^{\ln(\mathrm{ROR})\pm 1.96\sqrt{\frac{1}{a}+\frac{1}{b}+\frac{1}{c}+\frac{1}{d}}}$	a≥3 and lower limit of 95%CI >1
PRR	$\text{PRR}=\frac{\text{a}/\left(\text{a}+\text{b}\right)}{\text{c}/\left(\text{c}+\text{d}\right)}$ $95\%\mathrm{Cl}=e^{\ln(\mathrm{PRR})\pm 1.96\sqrt{\frac{1}{a}+\frac{1}{b}+\frac{1}{c}+\frac{1}{d}}}$	a≥3, PRR≥2 and lower limit of 95%CI >1
MHRA	${\text{χ}}^{2}=\frac{\text{n}{\left(\left|\text{ad}-\text{bc}\right|-\frac{\text{n}}{2}\right)}^{2}}{\left(\text{a}+\text{b}\right)\left(\text{a}+\text{c}\right)\left(\text{b}+\text{c}\right)\left(\text{c}+\text{d}\right)}$	a≥3 and χ2≥4

a: number of reports containing both the suspect drug and the suspect ADR;

b: number of reports containing the suspect ADR with other medications (except the drug of a);

c: number of reports containing the suspect drug with other ADRs (except the event of a);

d: number of reports containing other medications and other ADRs.

## Results

A total of 15,910 ADR reports related to antineoplastic drugs were collected in Henan Provincial Adverse Drug Reaction Monitoring Center from 2016 to 2020, of which 431 (2.71%) occurred in pediatric patients.

### Sample Characteristics

**Figure 1:**
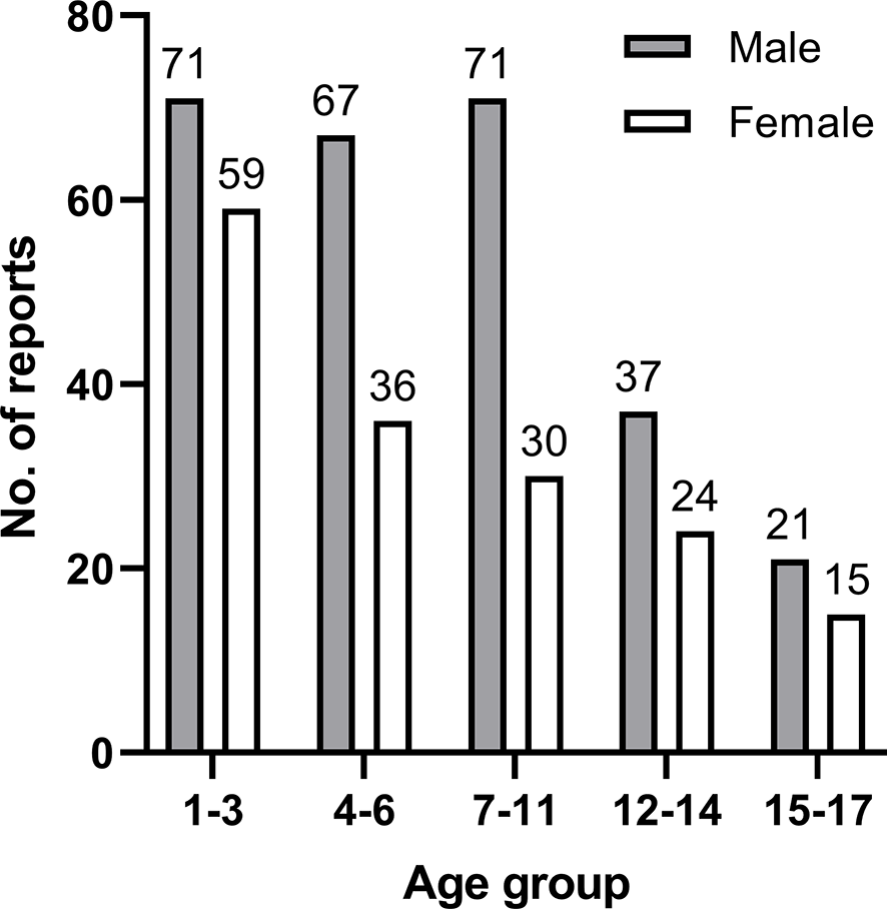
Number of reports in each age group by gender.

**Figure 2:**
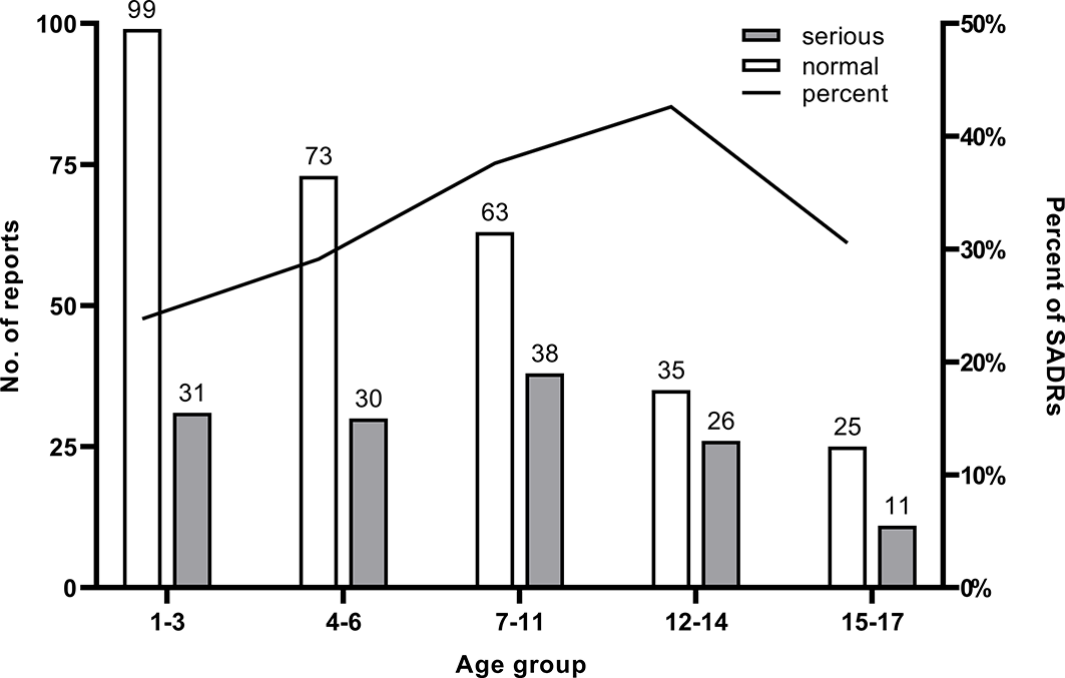
Number of reports and the proportion of SADR reports in each age group.

**Table 2** shows patient characteristics based on ADR severity. More ADRs were reported for boys than girls in every age group. No significant difference in the severity of ADRs was found between genders. The median age of patients was 6 years (inter quartile range, IQR: 3–11), and no report about patients younger than 1 was found. The gender differences in specific age groups were significant. The age groups with high reporting rates were concentrated in 1–3 years (130) and 4–6 years (103) (see **Figure 1**). **Figure 2** describes serious and normal reports in different age groups and the proportion of serious reports in each age group. Notably, the proportion of serious reports steadily increased with age, except in 15–17 year age group. Approximately 3.94% of patients suffered more than one disease before the ADRs occurred, and 10.44% had a history of ADR. Moreover, 29.7% of ADRs occurred 1–3 days after use. Approximately 79.8% of ADRs were reported within 1 week of medication, and only 1.62% of ADRs occurred after 1 month.

**Table 2:** The characteristics of patients aspect by severity of ADR.

Characteristic	Serious	Normal	*p* value^a^
	N = 136 (%)	N = 295 (%)	
**Gender**			
Male	88 (33.0)	179 (67.0)	0.423
Female	48 (29.3)	116 (70.7)	
**Age group (years)**			
1-3	31 (23.8)	99 (76.2)	0.060
4-6	30 (29.1)	73 (70.9)	
7-11	38 (37.6)	63 (62.4)	
12-14	26 (42.6)	35 (57.4)	
15-17	11 (30.6)	25 (69.4)	
**Disease types**			
Single	127 (31.3)	279 (68.7)	0.622
Multiple	9 (36.0)	16 (64.0)	
**Past history of ADR**			
Yes	22 (48.9)	23 (51.1)	0.008
No	114 (29.5)	272 (70.5)	
**Occurrence time of ADR (days)**			
On the day	30 (23.4)	98 (76.6)	<0.001^b^
1-3	31 (22.1)	109 (77.9)	
4-7	25 (32.9)	51 (67.1)	
8-14	42 (63.6)	24 (36.4)	
15-30	6 (42.9)	8 (57.1)	
Over a month	2 (28.6)	5 (71.4)	

^a^Chi-squared test; ^b^Fisher exact test.

### The Medication Characteristics

**Table 3** shows the ADR characteristics according to the severity. Difference in the proportion of polypharmacy was found (p < 0.001). Most patients received injection therapy (94.20%). Off-label drug use was not common in pediatric patients (7.42%).

**Table 3:** The characteristics of physicians aspect by severity of ADR.

Characteristic	Serious	Normal	*p* value^a^
	N = 136 (%)	N = 295 (%)	
**Polypharmacy (≥2 medications)**			
Polypharmacy	86 (40.4)	127 (59.6)	<0.001
Non-polypharmacy	50 (22.9)	168 (77.1)	
**Polypharmacy (≥5 medications)**			
Polypharmacy	19 (59.4)	13 (40.6)	<0.001
Non-polypharmacy	117 (29.3)	282 (70.7)	
**Route of administration**			
Parenteral	130 (32.0)	276 (68.0)	0.656^b^
Oral	3 (30.0)	7 (70.0)	
Others	3 (20.0)	12 (80.0)	
**Off-label** drug use			
Yes	8 (25.0)	24 (75.0)	0.407
No	128 (32.1)	271 (67.9)	

^a^Chi-squared test; ^b^Fisher exact test.

### The ADR Characteristics

**Table 4** shows drug characteristics according to the severity of ADR. The largest share of ADRs were reported for cytarabine (26.22% of the total reports), followed by asparaginase (12.76%). Doxorubicin, daunorubicin, and cytarabine had the most proportions of SADRs. A total of 431 events involved 18 system organ class (SOC) reports, mainly including gastrointestinal, blood and lymphatic system, and skin and subcutaneous tissue disorders. The majority of ADRs for each SOC were normal, except blood and lymphatic system disorders and cardiac disorders. According to the statistics, 69 ADRs were identified, which were concentrated in myelosuppression, rash, and vomiting.

**Table 4:** The characteristics of ADRs aspect by the severity.

Characteristic	Serious	Normal
	N = 136 (%)	N = 295 (%)
**Drugs (top 10)**		
Cytarabine	48 (42.5)	65 (57.5)
Asparaginase	11 (20.0)	44 (80.0)
Methotrexate	10 (20.4)	39 (79.6)
Pegaspargase	13 (35.1)	24 (64.9)
Cyclophosphamide	9 (30.0)	21 (70.0)
Etoposide	8 (36.4)	14 (63.6)
Vincristine sulfate	3 (17.6)	14 (82.4)
Cisplatin	4 (33.3)	8 (66.7)
Daunorubicin	5 (45.5)	6 (54.5)
Doxorubicin	6 (66.7)	3 (33.3)
**System organ class (top 10)**		
Gastrointestinal disorders	19 (14.6)	111 (85.4)
Blood and lymphatic system disorders	73 (70.9)	30 (29.1)
Skin and subcutaneous tissue disorders	15 (17.6)	70 (82.4)
General disorders and administration site conditions	10 (21.3)	37 (78.7)
Respiratory, thoracic, and mediastinal disorders	5 (41.7)	7 (58.3)
Nervous system disorders	1 (10.0)	9 (90.0)
Hepatobiliary disorders	4 (40.0)	6 (60.0)
Metabolism and nutrition disorders	0 (0)	7 (100)
Cardiac disorders	4 (57.1)	3 (42.9)
Immune system disorders	1 (20.0)	4 (80.0)
**ADR (top 10)**		
Myelosuppression	57 (85.1)	10 (14.9)
Rash	9 (15.0)	51 (85.0)
Vomiting	7 (13.7)	44 (86.3)
Fever	9 (24.3)	28 (75.7)
Nausea	3 (11.5)	23 (88.5)
Gastrointestinal reaction	2 (10.0)	18 (90.0)
Fibrinogen decreased	3 (25.0)	9 (75.0)
Leukocyte count decreased	6 (60.0)	4 (40.0)
Dyspnea	4 (44.4)	5 (55.6)
Hepatic failure	4 (44.4)	5 (55.6)

### Signal Mining

According to the calculation formulas and thresholds, DEC signals that did not meet the criteria were excluded. The three signal mining methods produced a total of 14 signals (see **Table 5**). The strength of the correlation between the drug and ADR increased with the ROR and PRR values.

**Table 5:** Signals of ADRs.

Drug	ADR	No.	ROR	95% CI Lower limit	PRR	95% CI Lower limit	χ^2^
Vincristine	Alopecia	4	63.39	10.64	48.71	10.90	327253.85
Asparaginase	Cyanosis^a^	3	21.64	2.21	20.51	2.22	114461.47
Pegaspargase	Itch^a^	3	17.29	2.79	15.97	2.82	94729.67
Methotrexate	Mucositis oral	7	63.50	7.63	54.57	7.69	42646.14
Asparaginase	Dyspnea	6	15.22	3.69	13.67	3.73	11950.02
Methotrexate	Hepatic failure	4	6.70	1.74	6.24	1.75	11208.07
Pegaspargase	Fibrinogen decreased	4	5.85	1.67	5.32	1.69	8295.83
Asparaginase	Fibrinogen decreased	8	15.83	4.59	13.67	4.65	5071.55
Cyclophosphamide	Reaction gastrointestinal	7	9.08	3.31	7.20	3.41	2756.44
Fludarabine	Myelosuppression	3	17.02	1.74	5.00	3.74	1256.66
Cisplatin	Vomiting	6	8.31	2.57	4.66	3.00	686.61
Etoposide	Rash^a^	7	3.14	1.22	2.46	1.29	236.91
Pegaspargase	Rash	13	4.00	1.91	2.95	2.01	73.08
Asparaginase	Rash	18	3.87	2.02	2.93	2.12	25.59

a Off-label ADR.

## Discussion

To the best of our knowledge, our study was the first to examine the safety profile of antineoplastic drugs in pediatric cancer patients in China on the basis of the data of Henan Provincial Adverse Drug Reaction Monitoring Center. Our study showed that approximately 2.71% (n = 431) of cancer patients’ reports were related to pediatric cancer patients. This percentage was in accordance with the result of the study in the Campania, south of Italy, where 3% of all individual case safety reports were found (11). Moreover, cancer is a rare disease in children, representing only 2% of all cancer cases (18).

SADRs seriously threaten the lives and health of patients and cause waste of medical resources. The China Adverse Drug Reaction Monitoring System has received 1.676 million ADR reports in 2020, and SADRs accounted for 10% of these reports (19). Our study found that the frequency of SADRs in children (31.55%) was higher than was generally reported in other pharmacovigilance studies. A retrospective analysis concerning children in Spanish Pharmacovigilance System observed 1419 ADRs, of which 4.4% were serious (20). Therefore, medical staff must carry out relevant health education to patients and their families to increase their knowledge about diseases, drugs, and ADRs.

Some pediatric studies found that a high proportion of reports about ADRs in males (21, 22). In our research, nearly three-fifths of the reports were related to males (n = 267). Due to the limitation of the database, the total number of patients using the drugs was unknown. According to the research of cancer incidence and mortality among children from 2010 to 2014 in Henan Province, China, the cancer incidence was predominant in boys, and the sex ratio was 1.19 (23). The difference in cancer incidence indicates that even if the number of reports differs between males and females, this does not mean that it is a gender difference. And no evidence of an association (p = 0.423) between gender and ADR severity was found. Further investigations are needed to explain this finding.

In our study, more than 50% of the ADRs were reported in children aged 1–6, and 30% of children are between 1 and 3. Although the proportion of SADRs increased with age, the overall number of ADR reports decreased with the age, and the result was not statistically significant. Because the data is collected from the spontaneous reporting system, it captures only a small fraction of the ADR that actually takes place (24). Due to the lack of consideration for the overall ADR incidence rate in the general population, the result only inflected the status of pediatric cancer patients who have been suffered from ADR in the region, or even more limited. Similar findings were observed in other studies (22, 25). Some reasons might have contributed to the high reporting rates in young children. First, immature tubular function reduces metabolism and liver function in young children, increasing the possibility of ADRs. Second, physicians and parents monitored young children closely because children lack expression ability, and thus ADRs were immediately found and reported in time. Physicians have to consider children’s sensitive physical conditions and the characteristics of chemotherapeutic agents when treating pediatric cancer patients.

The results showed that the proportion of pediatric patients with multiple disease was only 5.80%, identical to the expected result. Some studies in adults demonstrated that suffering from multiple diseases is a risk factor for SADRs (26), and might correlate with the decrease in drug metabolism or the damage to liver and kidney functions. Interaction among diseases might result in poor physical, emotional and social functions (27). However, in our study, children with multiple disease did not show significant differences in ADR severity.

The statistics showed that the severity of ADR in children who had ADR histories was significantly higher. Physicians should pay more attention to patients who have past histories of ADR and take caution when treating patients who are unsure about whether or not they have a history of drug allergies. This finding was also reflected in the reports. In 49.42% of these reports, physicians abandoned suspected drugs after ADRs occurred and did not continue using them.

In terms of the occurrence time of ADRs, 32.48% occurred 1–3 days after administration, and approximately 80% were found within 1 week. Early observation played a crucial role in pediatric patients. Family members should be reminded to monitor patients closely and continue to observe them for a week in order that ADRs can be detected and treated in time. Our results suggest evidence of an association (p<0.001) between occurrence time and the severity of an ADR. SADR was more common in chronic ADR from one week to one month, which means that long-term monitoring and follow-up of patients were desirable.

Polypharmacy is increasingly common, and a constant flow of novel therapeutic agents and treatment indications for existing medications has been observed (28, 29). The number of reported ADRs is expected to increase (22). Many studies showed the risk of SADR increases with the increase of the number of drugs (30, 31). Our results demonstrated that under two different definitions, the analysis showed significant association between polypharmacy exposure and SADR (p<0.001). Although many combinations of drugs have been found effective in relevant studies, formulating reasonable drug treatment strategies are still needed to minimize the risk of ADRs in children. In order to minimize the risk of SADRs, physicians should pay particular attention to children who are prescribed two or more drugs and closely monitor drug administration and (32).

Nearly all ADRs related to antineoplastic drugs occurred after injection given that drugs enter directly into the bloodstream and elicit reactions from the immune system. The safety of pediatric injection has been a concern. According to the Annual Report for National ADR Monitoring (2020) released by the China National Center of ADR Monitoring, more than 70% of ADRs in children are related to injections. As a special group, children are more sensitive to drugs and less tolerable because their organ development is incomplete. Thus, their risk of injection medication needs to be thoroughly examined.

Many medicines are prescribed to pediatric patients on an unlicensed or ‘off-label’ basis. Owing to the lack of adequately tested or authorized drugs, the use of off-label drugs exposes a child to a high risk of SADRs (33). The availability of medicines specifically designed for pediatric patients is limited, and less than 15% of currently marketed drugs specifically intended for children are operated on the basis of clinical trials. This reality is currently faced by many pediatricians (34). In our study, we found a low number of ADRs classified as off-label (7.42%). The proportion of SADR reports related to off-label drug use was low, but the existing evidence can not prove the reliability of off-label drug use. Among the off-label reports, most reports explored the use of vindesine for lymphocytic leukemia. In the literature, data on the efficacy and safety of vindesine for acute lymphocytic leukemia in children dated back to the 1980s, and attention should be paid to its clinical drug resistance (35, 36). The majority of ADRs observed in off-label cases belonged to gastrointestinal disorders and were mainly about myelosuppression and gastrointestinal reactions. In China, no relevant regulations on off-label drug use have been established. The China Food and Drug Administration (now called the NMPA) issued The Regulations on Drug Insert and Label in 2006. However, the regulation does not require specific information on pediatric populations and has not been revised (37). Thus, strengthening early assessment and risk management is imperative and crucial to the improvement of drug response and reduction of ADRs.

Our study showed that doxorubicin, daunorubicin, and cytarabine are the specific drugs with the most percentage reported associated with SADR. The frequency of cytarabine use was the largest, and the principal ADR was myelosuppression. We found that main indications for cytarabine as a myelosuppressive agent were used to treat acute myeloid leukemia. Cutaneous reactions were reported in the literature, and such events were found in our study (38). As for doxorubicin and daunorubicin, myelosuppressive and vomiting were reported (39, 40). Prevention and self-management education for patients and their families should be considered when ADRs are explained to them. This approach enables them to become familiar with the specific drugs they use.

The distribution of ADRs by system organ class was similar to the records in Italy (41), but more ADRs affected the blood and lymphatic system disorders in our study. Among the gastrointestinal disorders with the highest number of reports, chemotherapy-induced vomiting, nausea, and gastrointestinal reactions were the most common ADRs. The possible reason was that almost all drug regimens increased the risk of gastrointestinal system disorders, particularly cytarabine (42), methotrexate (43), and pegaspargase (44). SADRs accounted for the highest proportions of blood and lymphatic system disorders. Myelosuppression is the most common ADR and presents the most SADR reports in the study probably because of the prevalence of patients diagnosed with hematological tumors was the highest. Thus, strategies for monitoring, early detecting, confirming diagnosis, and providing appropriate supportive care are needed for hematological tumor therapy (45).

In the context of antineoplastic drugs in pediatric cancer patients, some common ADRs do not generate signals in data mining. By contrast, the ADRs of some drugs generated signals. This finding indicated that the ADRs and drugs were related. Combined with drug instructions, three off-label ADRs were found, including asparaginase-cyanosis, pegaspargase-itch, and etoposide-rash.

Cyanosis seemed to be an uncommon ADR of asparaginase. Few cases were found in related studies. Norman found that 16 cases of asparaginase had two cases of cyanosis and choking episodes, and these side effects were generally mild (46). However, the specific mechanism is unclear.

No report of itch caused by pegaspargase was found, but a study reported a pruritus ADR. One analysis consisted of eight individuals who had drug-induced liver injuries caused by asparaginase or pegaspargase, and only one patient mentioned itch (47). This finding may be attributed to metabolic status rather than to pharmacologic metabolism.

Many reports of rash when etoposide was used were found, which supported the results of this study. Mansfield found 65 cases of rash in 394 cases that used atezolizumab and carboplatin combined with etoposide (48). In-depth research should be performed to uncover their mechanisms of damage to the skin.

## Limitations

In China, ADR reports were reported by basic units (including drug manufacturers, pharmacies, and medical institutions) to provincial ADR monitoring centers. The reports are then evaluated by provincial and national ADR monitoring centers. The strict evaluation process ensures the accuracy of ADR reports but leads to a high rate of under-reporting. In our research, nearly all reports came from hospitals. A much higher number of ADRs may have occurred in real life. We encourage consumers and non-medical personnel to report ADRs to the system for the assessment of ADRs and reduction of bias.

Due to the limitation of the signal mining method and sample quantity, the results cannot represent the inevitable causal relationship between drugs and adverse reactions. The causal relationship, which includes the potential impact of false positives or negatives, needs further evaluation and verification. The methods we used — including ROR, PRR and MHRA — were frequentist statistical approaches, which means the limits of detecting false-positive signals and low specificity were unavoidable (49). When the number of reports is small, its stability will be greatly affected. Thus, signal mining aims to detect unknown ADR signals and provide more information and reference.

This study has several limitations. First, this study used the database of Henan Province, which does not necessarily represent the actual situation of the whole country. Second, because we selected one of the main adverse reactions from one report, the potential relationship between ADR and drugs may not be explored. More research is needed to confirm the possible potential relationship. Third, subject to the spontaneous reporting system and the database, our research has some limitations, such as underreporting, unstable reporting, not getting incidence rates, and difficulty determining causality. Further studies using larger databases are needed to evaluate ADRs in greater detail.

## Conclusion

The incidence of ADRs in pediatric cancer patients during therapy was high, with different features involving various systems organs class. Most ADRs were normal in severity, while some were serious. These ADRs were mostly acute and occurred within one week of administration. Age, past history of ADR, occurrence time of ADR, and polypharmacy may be relative to the severity of ADRs. Signal mining produced 14 signals. Three of them were off-label ADR. Therefore, the characteristics of ADRs obtained in this study could accumulate experience for clinical staff to carry out ADRs management to ensure the safety of pediatric patients. More researches are needed to achieve a better understanding of the characteristics of ADRs in pediatric cancer patients of China.

## Data Availability Statement

The datasets for this article are not publicly available because the pharmacovigilance data in single, non-aggregated form are available only under a specific authorization released by the Medical Products Administration and Center for ADR Monitoring of Henan. Requests to access the datasets should be directed to the Medical Products Administration and Center for ADR Monitoring of Henan.

## Ethics Statement

The studies involving human participants were reviewed and approved by the Ethics Committee of Tongji Medical College, Huazhong University of Science and Technology (2020S204). Written informed consent to participate in this study was provided by the participants’ legal guardian/next of kin.

## Author Contributions

All authors were involved in the conception and design of the study, interpretation of data, and drafting or revising the manuscript critically for important intellectual content.

## Funding

This work was supported by the National Youth Natural Science Foundation of China (No. 71804052), the National Natural Science Foundation of China (No. 72074088) and the Health Commission of Hubei Province Fund (Grant Number: WJ2021Q022).

## Conflict of Interest

The authors declare that the research was conducted in the absence of any commercial or financial relationships that could be construed as a potential conflict of interest.

## Publisher’s Note

All claims expressed in this article are solely those of the authors and do not necessarily represent those of their affiliated organizations, or those of the publisher, the editors and the reviewers. Any product that may be evaluated in this article, or claim that may be made by its manufacturer, is not guaranteed or endorsed by the publisher.
