# Corrigendum: Suspected Adverse Drug Reactions in Pediatric Cancer Patients in China: An Analysis of Henan Province Spontaneous Reporting System Database

**DOI:** 10.3389/fonc.2022.859596

**Published:** 2022-02-10

**Authors:** Zhiming Jiao, Zhanchun Feng, Ziqi Yan, Jinwen Zhang, Gang Li, Ganyi Wang, Qianyu Wang, Da Feng

**Affiliations:** ^1^ School of Medicine and Health Management, Tongji Medical College, Huazhong University of Science and Technology, Wuhan, China; ^2^ Department of Pharmacy, Tongji Hospital, Tongji Medical College, Huazhong University of Science and Technology, Wuhan, China; ^3^ Medical Products Administration and Center for Adverse Drug Reaction (ADR) Monitoring of Henan, Zhengzhou, China; ^4^ College of Public Administration, Huazhong University of Science and Technology, Wuhan, China; ^5^ School of Pharmacy, Tongji Medical College, Huazhong University of Science and Technology, Wuhan, China

**Keywords:** pharmacovigilance, drug monitoring, adverse drug reaction reporting systems, pediatric, medical oncology

There is an error in the Funding statement. The correct number for the National Youth Natural Science Foundation of China is 71804052.

The authors apologize for this error and state that this does not change the scientific conclusions of the article in any way. The original article has been updated.

## Publisher’s Note

All claims expressed in this article are solely those of the authors and do not necessarily represent those of their affiliated organizations, or those of the publisher, the editors and the reviewers. Any product that may be evaluated in this article, or claim that may be made by its manufacturer, is not guaranteed or endorsed by the publisher.

